# Physiological levels of estradiol limit murine osteoarthritis progression

**DOI:** 10.1530/JOE-22-0032

**Published:** 2022-08-16

**Authors:** Carmen Corciulo, Julia M Scheffler, Piotr Humeniuk, Alicia Del Carpio Pons, Alexandra Stubelius, Ula Von Mentzer, Christina Drevinge, Aidan Barrett, Sofia Wüstenhagen, Matti Poutanen, Claes Ohlsson, Marie K Lagerquist, Ulrika Islander

**Affiliations:** 1 Centre for Bone and Arthritis Research, Department of Rheumatology and Inflammation Research, Institute of Medicine, Sahlgrenska Academy at the University of Gothenburg, Gothenburg, Sweden; 2 Sahlgrenska Osteoporosis Centre, Centre for Bone and Arthritis Research, Department of Internal Medicine and Clinical Nutrition, Institute of Medicine, Sahlgrenska Academy at the University of Gothenburg, Gothenburg, Sweden; 3 Division of Chemical Biology, Department of Biology and Biological Engineering, Chalmers University of Technology, Gothenburg, Sweden; 4 Institute of Biomedicine, Research Centre for Integrative Physiology and Pharmacology, Turku Center for Disease Modeling, University of Turku, Turku, Finland; 5 Department of Drug Treatment, Sahlgrenska University Hospital, Gothenburg, Sweden

**Keywords:** estradiol, osteoarthritis, cartilage, T cells, bone

## Abstract

Among patients with knee osteoarthritis (OA), postmenopausal women are over-represented. The purpose of this study was to determine whether deficiency of female sex steroids affects OA progression and to evaluate the protective effect of treatment with a physiological dose of 17β-estradiol (E2) on OA progression using a murine model. Ovariectomy (OVX) of female mice was used to mimic a postmenopausal state. OVX or sham-operated mice underwent surgery for destabilization of the medial meniscus (DMM) to induce OA. E2 was administered in a pulsed manner for 2 and 8 weeks. OVX of OA mice did not influence the cartilage phenotype or synovial thickness, while both cortical and trabecular subchondral bone mineral density (BMD) decreased after OVX compared with sham-operated mice at 8 weeks post-DMM surgery. Additionally, OVX mice displayed decreased motor activity, reduced threshold of pain sensitivity, and increased number of T cells in the inguinal lymph nodes compared to sham-operated mice 2 weeks after OA induction. Eight weeks of treatment with E2 prevented cartilage damage and thickening of the synovium in OVX OA mice. The motor activity was improved after E2 replacement at the 2 weeks time point, which was also associated with lower pain sensitivity in the OA paw. E2 treatment protected against OVX-induced loss of subchondral trabecular bone. The number of T cells in the inguinal lymph nodes was reduced by E2 treatment after 8 weeks. This study demonstrates that treatment with a physiological dose of E2 exerts a protective role by reducing OA symptoms.

## Introduction

Osteoarthritis (OA) is a debilitating disease characterized by degenerative processes of the articular cartilage that are exacerbated by mild local inflammation (Robinson *et al.* 2016). Erosion of the joint cartilage is associated with structural subchondral bone damage, increased pain sensitivity also in body parts that are not directly affected by the disease, and mild inflammation of the synovium (Loeser *et al.* 2012).

OA affects more than 240 million people globally and represents a growing social problem due to the important repercussions on the economy and the life of the patients. Pain, disability, and social isolation lead to a drastic decline in the quality of life for OA patients (Nelson 2018, Hawker 2019, Allen *et al.* 2022). Results from a substantial amount of studies on OA are now available, with an important contribution from preclinical models that compensate for the limited access to human OA joint tissue at an early stage of the disease (Vincent 2020).

Nevertheless, the complex mechanisms underlying OA pathology are still not completely understood and therapies able to revert the disease progression have not been identified. However, it is well known that the female sex represents a significant risk factor.

Among OA patients over the age of 40, postmenopausal women are over-represented, with a sex difference in the incidence of knee OA ranging from 46% in women to 21% in men (Prieto-Alhambra *et al.* 2014, Hunter & Bierma-Zeinstra 2019). Epidemiological studies associate menopause with articular cartilage degeneration, OA severity, and unsuccessful joint replacement. The decline of sex steroids after menopause, in particular estrogens, has been investigated as responsible for the triggering of the disease (Talsania & Scofield 2017, Hunter & Bierma-Zeinstra 2019). Estrogen receptors (ERs), ERα and ERβ, have been found in several tissues of the joint (Capellino *et al.* 2007, Emmanuelle *et al.* 2021, Tang *et al.* 2021). Moreover, estradiol is a well-known regulator of bone and immune system homeostasis and also controls motor ability and pain sensitivity (Straub 2007). Although epidemiological studies reported that postmenopausal women have a higher risk to develop OA compared to men, the association between the risk to develop OA and hormonal factors is not clear (de Klerk *et al.* 2009). The effect of ovariectomy (OVX) on experimental OA models consistently points toward the deterioration of the articular cartilage, while the effect of estradiol replacement displays conflicting results (Roman-Blas *et al.* 2009). The discrepancy is largely dependent on the experimental setup, including dose, frequency, and route of administration of the steroid hormone (Turner *et al.* 1997, Rasanen & Messner 1999, Christgau *et al.* 2004, Sniekers *et al.* 2008, 2010).

The purpose of this study was to clarify the role of female sex steroid deficiency in OA progression and to determine the effect of treatment with a physiological dose of estradiol administered in a pulsed manner, on cartilage and bone alterations, inflammation, and impairment of motor ability and pain sensitivity.

## Materials and methods

### Animals

**Figure 1:**
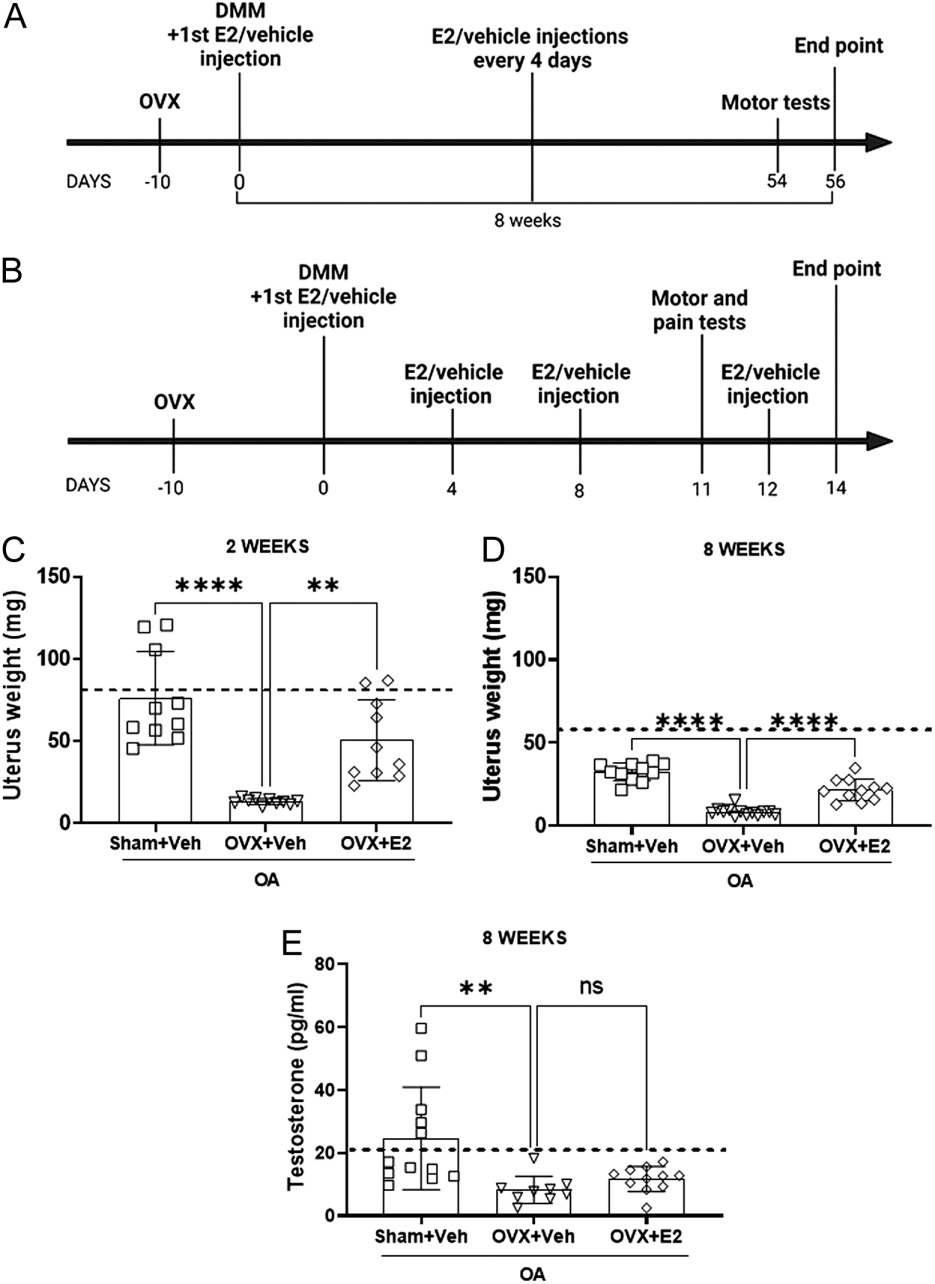
Experimental plan, uterus weight, and testosterone measurement. The schemes describe the timelines of the experiments including the 8 weeks (A) and 2 weeks E2 treatment experiments (B). Sham-operated and ovariectomized (OVX) mice were subjected to DMM surgery and treated with either vehicle (Veh) or 17β-estradiol (E2; 0.15 µg) every 4 days for 2 (A) and 8 weeks (B). Uterus weights confirm successful ovariectomy surgery and estradiol replacement after 2 weeks (C) and 8 weeks (D) of treatment. The amount of testosterone was measured in serum after 8 weeks of treatment (E). The dashed line indicates the mean value of the control group shown in Supplementary Fig. 1. Data are expressed as mean ± s.d. and analyzed by one-way ANOVA followed by Dunnet’s* post hoc* test. ns = not statistically significant, **P < 0.01, ****P < 0.0001.

Female C57BL/6J mice (Taconic, Borup, Denmark) were kept in the animal facility at the University of Gothenburg (Sweden) under regular lighting conditions (12 h light/12 h darkness cycle), fed soya-free laboratory chow and tap water *ad libitum*. Mice were acclimatized for 7 days before initiating the surgical procedures. The experiments were carried out following the timelines described in Fig. 1A and B. All the experimental procedures were performed in accordance with the ethical permit (2814-2020) approved by the Regional Ethical Review Board in Gothenburg, Sweden.

### Surgical procedures and 17β-estradiol treatment

Female mice, 8 weeks old, were employed in the study, and all underwent OVX or sham surgery as previously described (Corciulo *et al.* 2021). Mice were allowed to recover from the OVX procedure for 10 days before the surgery for destabilization of the medial meniscus (DMM). Mice were anesthetized, the right knee was shaved, and the area was sterilized with chlorhexidine solution. A 1-cm-long incision was made longitudinally on the medial area of the knee to expose the joint. The joint capsule was cut and the patella was dislocated to allow the excision of the menisco-tibial ligament. The wound on the skin was closed with silk sutures. Buprenorphine (0.1 mg/kg) was injected intraperitoneally as a postoperative analgesic. The control group included animals that underwent control surgery for DMM in which the menisco-tibial ligament was visualized but not transected.

An oil-based stock solution of E2 (17β-estradiol-3-benzoate, Sigma-Aldrich; 1 mg/mL) was prepared by mixing E2 with inert miglyol oil (Miglyol812, OmyaPeralta GmbH, Hamburg, Germany). E2 was dissolved by stirring the solution for 3 h at 150°C and then further diluted to 1.5 µg/mL in miglyol before the injection. Mice received the first s.c. injection (100 µL) of E2 (0.15 µg E2/mouse/injection) or vehicle (Veh) immediately after the DMM procedure. The subsequent doses were injected every 4 days to mimic the estrus cycle in mice.

### Experimental groups

The mice in this study were divided into four groups and included in the same experiment:

1. Control surgery for DMM; sham-operated; injections with Veh.
2. DMM surgery; sham-operated; injections with Veh.
3. DMM surgery; OVX; injections with Veh.
4. DMM surgery; OVX; injections with E2.

In the first set of analyses, groups 1 and 2 were compared in order to determine the severity and phenotype of the OA disease. The results are presented in Supplementary Figs 3, 4, 5, 6, 7, and 8 (see section on supplementary materials given at the end of this article). Group 1 is named ‘Control’ and group 2 is named ‘OA’ in the figures.

**Figure 2:**
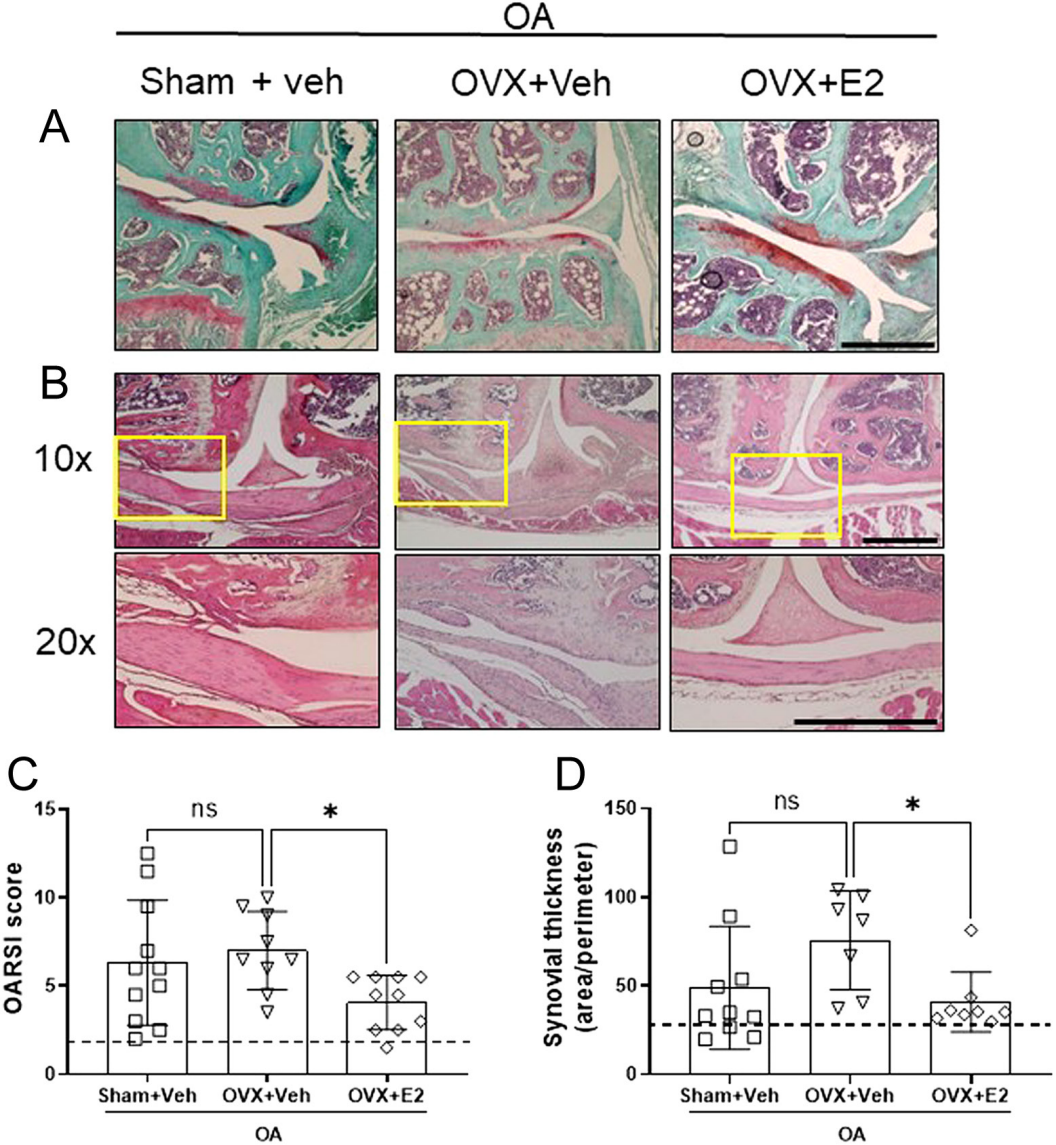
E2 replacement prevents cartilage degradation and hyperplasia of the synovial membrane in mice with OA. Sham-operated and ovariectomized (OVX) mice were subjected to destabilization of the medial meniscus (DMM) surgery and treated with either vehicle (Veh) or 17β-estradiol (E2; 0.15 µg) every 4 days for 8 weeks. Representative images of the knee articular surfaces stained with the Safranin-O/fast green (A, scale bar = 500 µm). Representative figures of hematoxylin/eosin staining of the knee joint (B, 10× magnification on the top section, 20× magnification on the bottom section; scale bar = 500 µm). The graphs show the plotted OARSI (Osteoarthritis Research Society International) score quantification (C) and the quantification of the synovial thickness (synovial area/perimeter; D). The dashed line indicates the mean value of the control group shown in Supplementary Fig. 2. Data are expressed as mean ± s.d. and analyzed by one-way ANOVA followed by Dunnet’s* post hoc* test. *P < 0.05, ns = not statistically significant.

**Figure 3:**
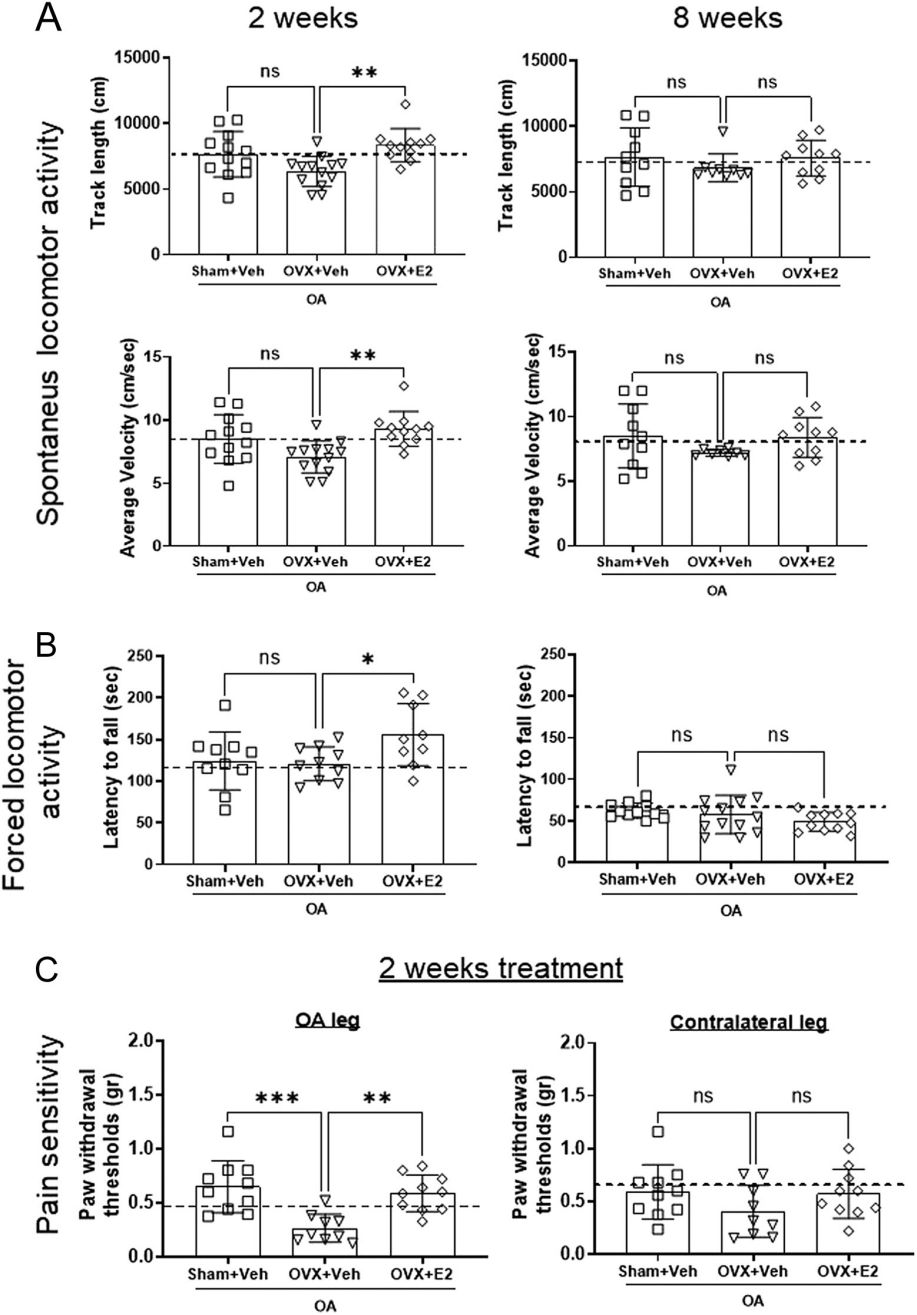
E2 treatment for 2 weeks improves the motor ability and pain sensitivity in mice with OA. Sham-operated and ovariectomized (OVX) mice were subjected to destabilization of the medial meniscus (DMM) surgery and treated with either vehicle (Veh) or 17β-estradiol (E2; 0.15 µg) every 4 days for 2 or 8 weeks. The graphs show the track length and the velocity of the experimental mice in the arena of the open field test (A) and the latency to fall from the rotarod apparatus (B). The von Frey test shows the paw withdrawal threshold for the OA leg and the contralateral leg (C). The dashed line indicates the mean value of the control group shown in Supplementary Fig. 3. Data are expressed as mean ± s.d. and analyzed by one-way ANOVA followed by Dunnet’s* post hoc* test. *P < 0.05; **P < 0.01; ***P < 0.001, ns = not statistically significant.

**Figure 4:**
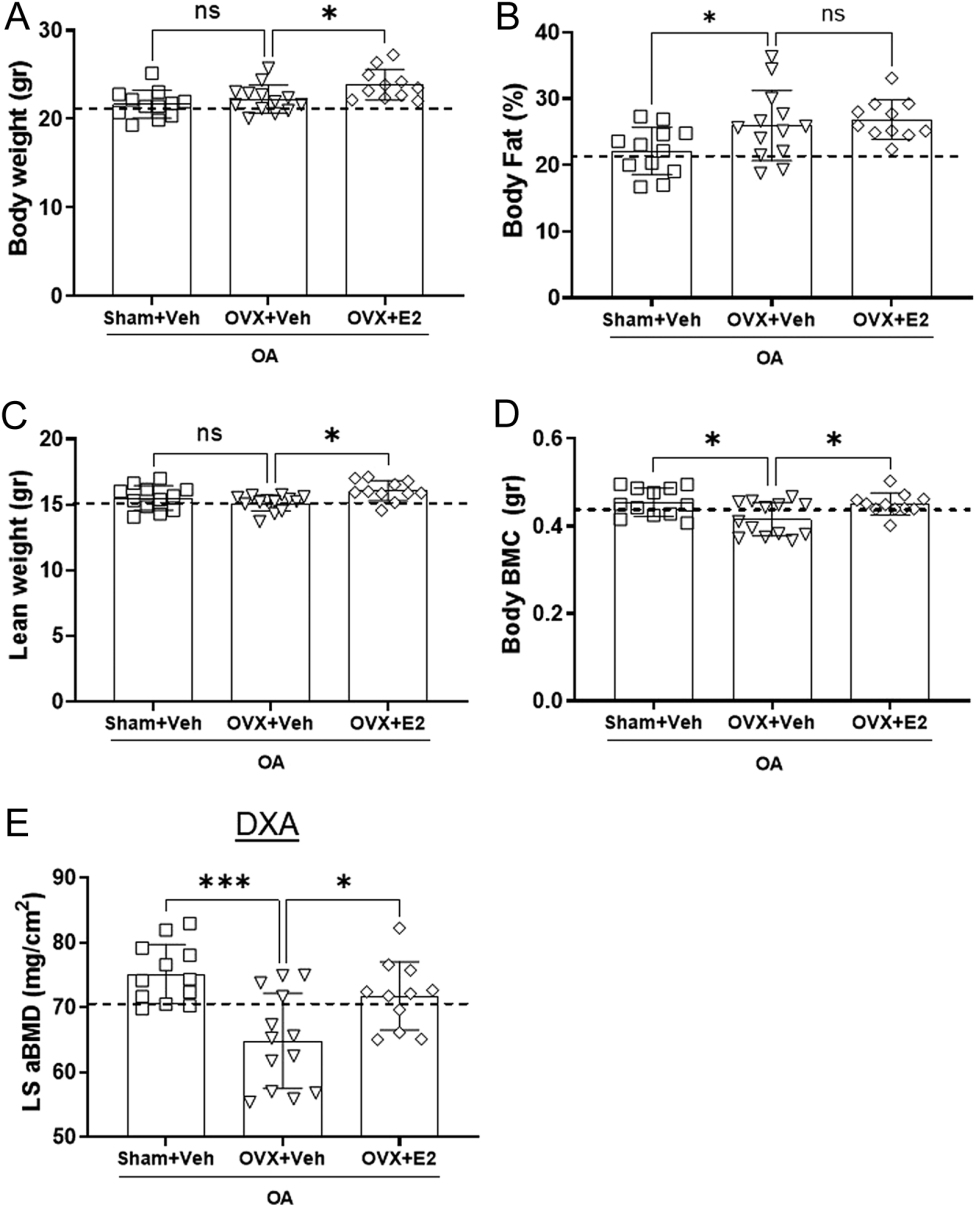
OVX and E2 treatment partly change the body composition of OA mice. Sham-operated and ovariectomized (OVX) mice were subjected to destabilization of the medial meniscus (DMM) surgery and treated with either vehicle (Veh) or 17β-estradiol (E2; 0.15 µg) every 4 days for 8 weeks. The graphs describe the total body weight (A), DXA measurements of the body fat percentage (B), lean weight (C), total body bone mineral content (BMC) (D), and areal bone mineral density (aBMD) of the lumbar spine (LS) (E). The dashed line indicates the mean value of the control group shown in Supplementary Fig. 4. Data are expressed as mean ± s.d. and analyzed by one-way ANOVA followed by Dunnet’s* post hoc* test. *P < 0.05, ***P < 0.001, ns = not statistically significant.

**Figure 5:**
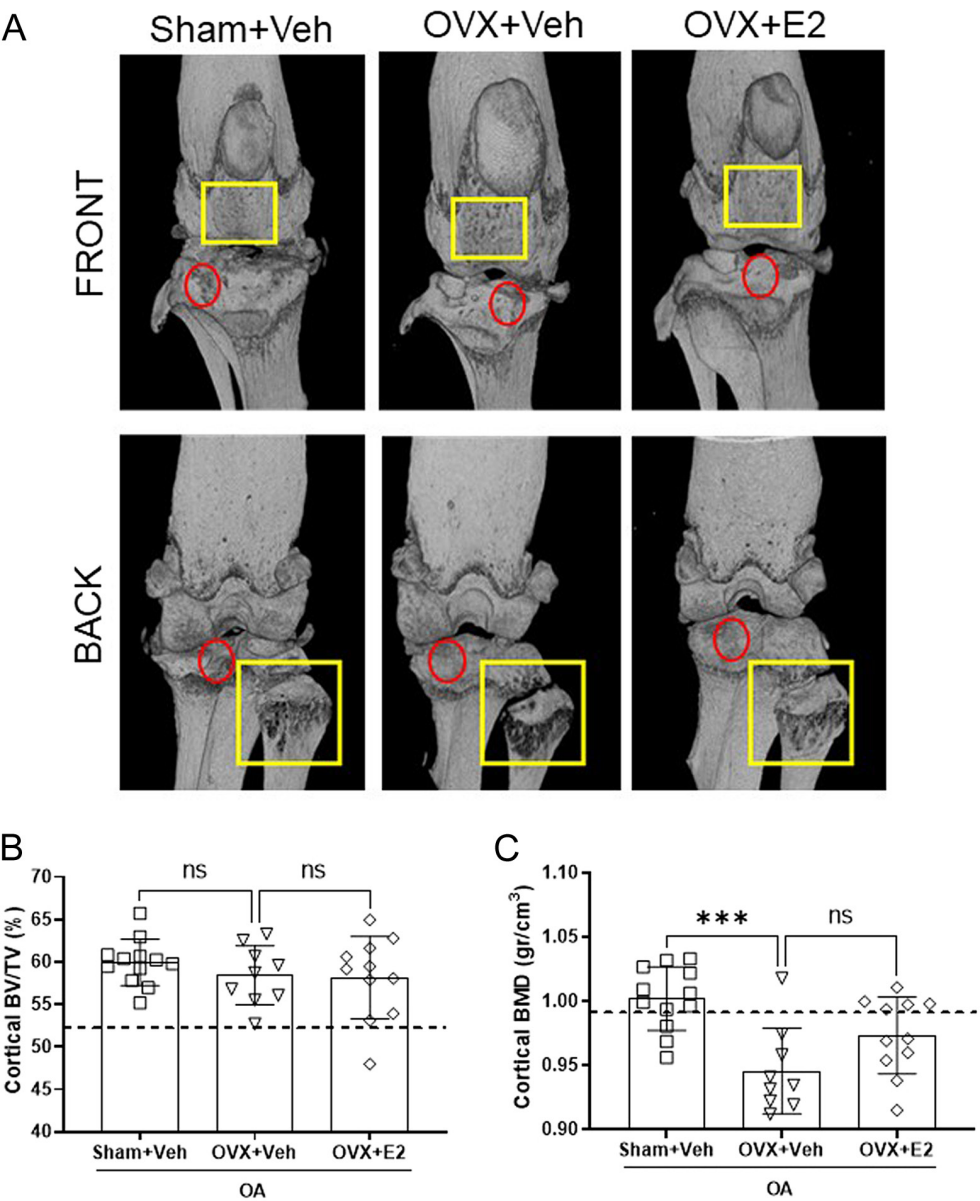
E2 treatment does not affect the cortical bone of OA mice. Sham-operated and ovariectomized (OVX) mice were subjected to destabilization of the medial meniscus (DMM) surgery and treated with either vehicle (Veh) or 17β-estradiol (E2; 0.15 µg) every 4 days for 8 weeks. Representative pictures of 3D knee joint reconstruction from µCT data (A). The yellow rectangles highlight the areas of bone loss and pittings of the articular surface. The red circles define the area where the irregular articular bone surface is visible. The graphs contain the plotted data from the analysis of the µCT data for the cortical bone volume/tissue volume (BV/TV; B) and the cortical bone mineral density (BMD; C). The dashed line indicates the mean value of the control group shown in Supplementary Fig. 5. Data are expressed as mean ± s.d. and analyzed by one-way ANOVA followed by Dunnet’s* post hoc* test. ***P < 0.001, ns = not statistically significant. A full color version of this figure is available at https://doi.org/10.1530/JOE-22-0032.

**Figure 6:**
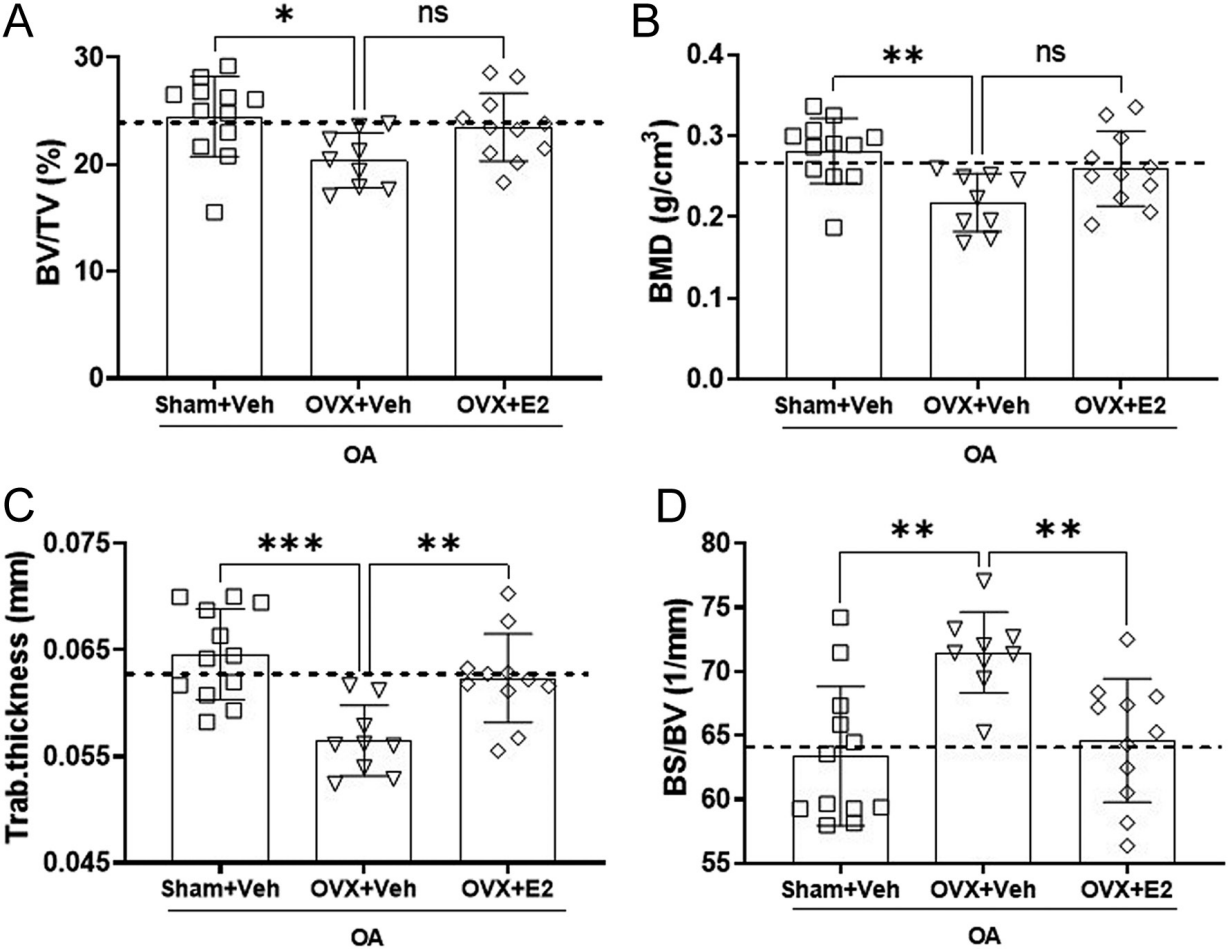
E2 treatment limits the subchondral trabecular bone loss in OVX OA mice. Sham-operated and ovariectomized (OVX) mice were subjected to destabilization of the medial meniscus (DMM) surgery and treated with either vehicle (Veh) or 17β-estradiol (E2; 0.15 µg) every 4 days for 8 weeks. The graphs display the plotted data from the analysis of the µCT data for the trabecular subchondral bone volume/tissue volume (BV/TV; A), trabecular bone mineral density (BMD; B), trabecular (Trab.) thickness (C), and bone surface/bone volume (bone erosion; D). The dashed line indicates the mean value of the control group shown in Supplementary Fig. 6. Data are expressed as mean ± s.d. and analyzed by one-way ANOVA followed by Dunnet’s* post hoc* test. *P < 0.05, **P < 0.01, ***P < 0.001, ns = not statistically significant.

**Figure 7:**
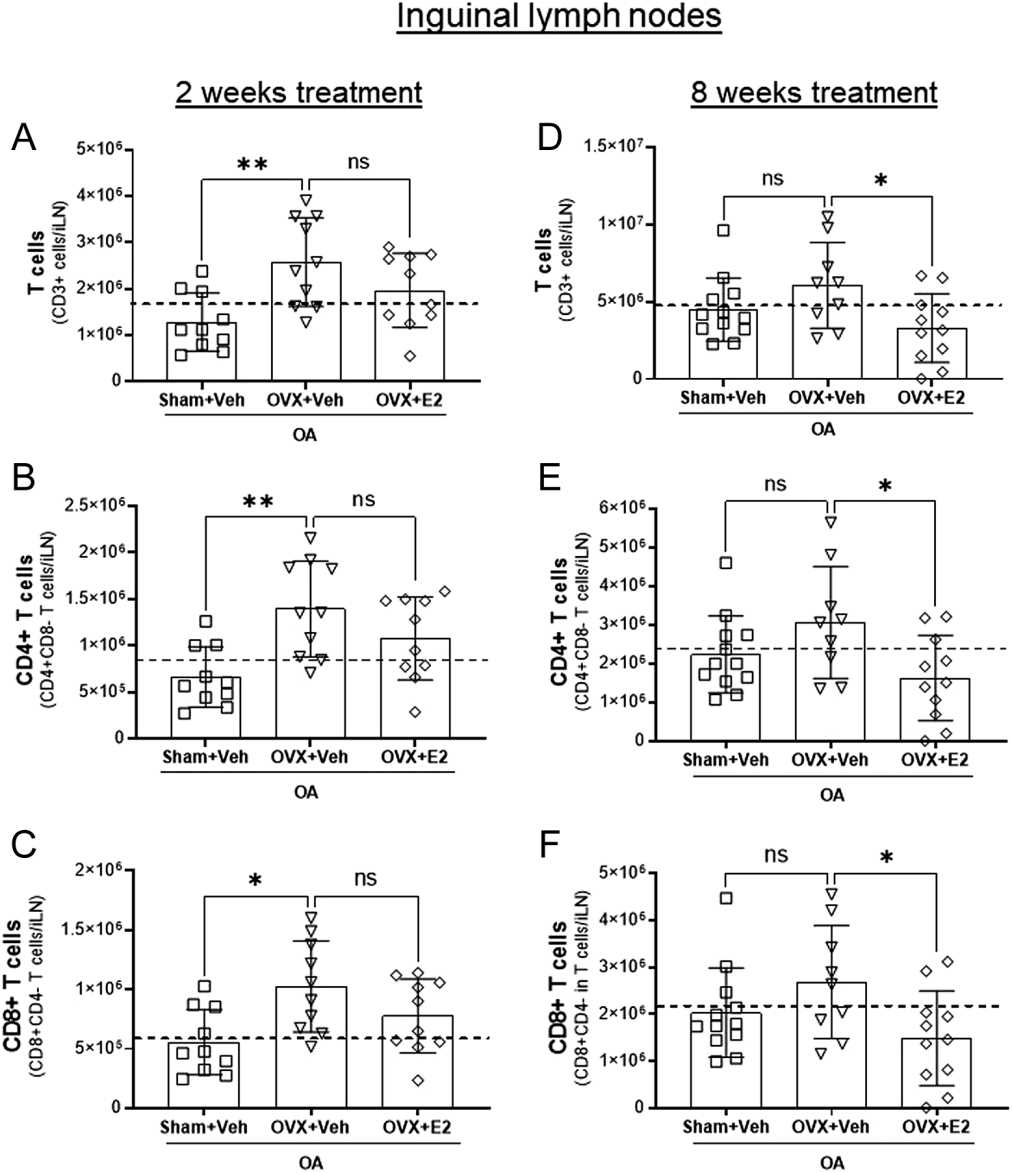
OVX and E2 treatments influence the number of CD4+ T cells and CD8+ T cells in inguinal lymph nodes of OA mice. Sham-operated and ovariectomized (OVX) mice were subjected to destabilization of the medial meniscus (DMM) surgery and treated either with vehicle (Veh) or 17β-estradiol (E2; 0.15 µg) every 4 days for 8 weeks. The graphs show the plotted data from the flow-cytometry analysis of total T cells, CD4+ T cells, and CD8+ T cells after 2 weeks (A–C) and 8 weeks of E2 treatment (D–F). The dashed line indicates the mean value of the control group shown in Supplementary Fig. 7. Data are expressed as mean ± s.d. and analyzed by one-way ANOVA followed by Dunnet’s* post hoc* test. *P < 0.05; **P < 0.01, ns = not statistically significant.

In the second approach, the three OA groups (groups 2–4, all subjected to DMM) were compared to evaluate the role of female sex steroid deficiency (OVX) and E2 replacement on OA progression. These results are presented in Figs 1, 2, 3, 4, 5, 6, and 7. Group 2 is named ‘Sham+Veh’, group 3 is ‘OVX+Veh’, group 4 is named ‘OVX+E2’. Thus, group 2 is used in both analyses.

### Assessment of motor ability and pain sensitivity

To assess the locomotor ability and pain sensitivity of the experimental animals, 2 different motor tests and the von Frey test for determination of pain sensitivity were performed 2 days before the experiment end-point. Mice were acclimatized in the procedure room for 1 h before the beginning of the tests.

Spontaneous locomotor activity was analyzed using the open-field test. Each mouse was placed in the center of a 60 × 60 × 60 cm chamber to allow free exploration. The experiments were performed for 15 min. The motor parameters were measured by computerized analysis. Mouse behavior was recorded and videos were analyzed using Viewer software (Biobserve, Sankt Augustin, Germany).

Forced locomotor activity was tested using the rotarod test. Mice were placed on a rotarod apparatus (Panlab, Harvard Apparatus, Cornella, Spain) and tested for 5 min with constantly increasing acceleration from 4 to 40 rpm. The latency to fall was registered for each animal. To exclude differences in learning skills between the groups of mice, each group was assessed over 3 trials per day for 2 consecutive days. The mice were given 30-min intertrial rest interval.

Von Frey filament stimulus-limb withdrawal test was performed to assess pain sensitivity on both hind paws of the mice included in the 2 weeks treatment experiment. The mice were placed in a clear plastic cage on top of a custom-manufactured metal mesh platform allowing full access to all paws. The mice were allowed to acclimatize to their surroundings for at least 30 min. The target for stimulation was the mid-plantar left hind paw, with a set of calibrated von Frey filaments of increasing stiffness (0.004–8 g) presented perpendicularly to the paw. In the process of the experimental procedure, the mechanical stimulation intensity increased and the behavior of the mouse was observed. Withdrawal of the hind limb upon introduction or immediately upon the removal of the filament were considered positive responses. The procedure was repeated 5 times every 30 s, and the head withdrawal threshold was recorded.

### Termination of the experiments and tissue collection

Mice were anesthetized with a mixture of ketamine/dexmedetomidine hydrochloride, and body composition was determined using a dual-energy x-ray absorptiometry (DXA) scan (UltraFocus^DXA^, Faxitron Bioptics, Tucson, AZ, USA). Anesthetized mice were euthanized by exsanguination followed by cervical dislocation. Uteri were dissected and weights were noted (Fig. 1A, B and Supplementary Fig. 1A, B). Inguinal lymph nodes (iLN) were collected for flow cytometry analysis. Knee joints and lumbar vertebrae were collected for micro-CT (µCT) analysis.

### Testosterone measurements in serum

Peripheral blood samples from all mice were collected at termination in 500 µL tubes containing serum gel with a clotting activator (Microvette 500 Z-Gel, Sarstedt, Numbrecht, Germany). The serum was extracted and stored at −80°C until use. Steroids were extracted from serum (200 µL) and concentrations of testosterone were analyzed by liquid chromatography-mass spectrometry as previously described (HPLC-MS; Acquity UPLC system and TQ-XS triple quadrupole mass spectrometer) (Ohlsson *et al.* 2022).

### Flow cytometry analysis

Lymph nodes were transferred to a meshed-cap vial (35 µm mesh), pressed through it with 400 µL of PBS and counted using an automated cell counter (Sysmex, Norderstedt, Germany). Cells were resuspended in FACS buffer (FBS 2%, EDTA 2 mM in PBS) and stained with eBioscience Fixable Viability Dye eFluor 780 (Thermo Fisher Scientific), followed by incubation with Fc-gamma receptor block (Becton Dickinson). The cells were then stained with fluorochrome-conjugated antibodies: CD3-BV510, CD4-A488 or CD4-APC, CD8-FITC or CD8-BV421 (BioLegend, San Diego, CA, USA). The cells were immediately acquired using FACSVerse (Becton Dickinson) and the data were analyzed with FlowJo software Version 10 (FlowJo_v10.6.1, Ashland, OR, USA). Forward and side scatter gates were used to discriminate doublets and debris (FSC-A, FSC-H, SSC-A × SSC-H). Fluorescence minus one was used as control. Only viable cells were included in the analysis.

### Micro-computed tomography

After sacrifice, the right hind leg was excised and the soft tissue was carefully removed from the bone. Samples were fixed in 4% paraformaldehyde for 3 days and then stored in 70% ethanol. The subchondral area for the trabecular bone evaluation starts below the cortical bone in the epiphyseal region and extends for a longitudinal distance of 495 µm in the distal direction. The region of interest (ROI) for the measurement of the subchondral cortical bone was defined as the 742 µm area extending in the distal direction starting from the tibial plateau. The ROI for the lumbar vertebrae L3 was defined as the 641 µm area starting at 1.296 mm in the caudal direction. The selected area was evaluated in a scanning tube providing a voxel size of 4.49 µm isotropically and scanned at 50 kV, 200 µA (Skyscan 1172 scanner; Bruker MicroCT, Kontich, Belgium). The samples were kept on paper soaked in PBS to avoid dehydration. Analysis of the morphology and measurement of bone features by μCT was performed using the software CtAN (1.13.2.1, Bruker microCT). A 3D reconstruction of the knee joint was performed by using CTvox (2.5.0 r892, Bruker microCT).

### Histology

After the µCT analysis, the knees were decalcified in 10% EDTA (Sigma-Aldrich) for 21 days at 4°C. Specimens were embedded in paraffin blocks and 5-µm coronal sections of the knees were obtained. The sections were used for hematoxylin/eosin (H&E) staining and Safranin-O/fast green staining.

Pictures of the slides were taken using the EVOS XL core microscope (Thermo Fisher Scientific). Assessment of OA was performed by evaluation of Safranin-O stained slides in a blinded fashion by two observers. OARSI score was determined blindly as previously described (Glasson *et al.* 2010). Briefly, for histologic scoring, slides were stained using the Safranin-O fast green technique. The OA severity was determined by using a 0–6 scoring system: 0 for normal cartilage; 0.5 in case of loss of Safranin-O without structural changes; 1 for small fibrillation without loss of cartilage; 2 when vertical clefts were present; 3, 4, 5, and 6 when vertical clefts and erosion covered <25%, 25–50%, 50–75%, and >75% of the articular surface, respectively.

### Data analysis

Gaussian distribution was assumed for all the data, and results are expressed as mean ± s.d. Statistically significant differences between groups were determined using Student’s t-test or one-way ANOVA followed by Dunnet’s* post hoc* test, as appropriate. Analyses were performed using GraphPad Prism software version 9 (GraphPad, CA, USA).

## Results

### OA mice display cartilage degradation and increased subchondral cortical bone at 8 weeks post-DMM surgery without effects on T cell numbers, motor activity, or pain threshold

The OA phenotype developing after the DMM surgery was analyzed by histologic Safranin-O staining of the knee followed by assessment of the OARSI score at 8 weeks post-surgery. Cartilage degeneration in OA mice was visible as loss of proteoglycans and fibrillations on the articular surface that lead to an increased OARSI score compared to control mice (Supplementary Fig. 2A and B). Synovial hyperplasia was determined by measuring the synovial thickness following H&E staining. No difference in synovial thickness was detected between the control and OA mice at 8 weeks post-DMM surgery (Supplementary Fig. 2C and D).

OA and control mice were subjected to spontaneous and forced locomotor activities at 2 and 8 weeks post-DMM surgery. No differences in motor abilities were detected between the groups at either time-point (Supplementary Fig. 3A and B). At 2 weeks post-surgery, no differences in the pain threshold were detected between OA and control mice (Supplementary Fig. 3C).

Furthermore, OA mice did not differ from control mice in body weight, fat and lean mass, and bone mineral content (BMC) measured by DXA after 8 weeks (Supplementary Fig. 4A, B, C, and D).

DXA scanning of the lumbar vertebrae (L3 and L4) showed increased areal bone mineral density (aBMD) in OA mice compared to controls (Supplementary Fig. 4E).

The 3D reconstruction of the µCT scanning showed alterations of the articular surface in both femur and tibia of OA mice compared to controls. The loss of bone in the joint is visible as bone pitting on the femur and loss of density on the tibia in OA mice compared to the control (Supplementary Fig. 4A). Quantification of the µCT scanning showed an increase of cortical bone volume/tissue volume (BV/TV) in the subchondral area of the tibia in OA mice compared to the control group (Supplementary Fig. 5B), in accordance with the development of subchondral bone sclerosis in the OA mice (Li *et al.* 2013). However, the cortical BMD did not change between the experimental groups (Supplementary Fig. 5C).

The trabecular bone volume in the tibial subchondral area did not differ between OA and control mice (Supplementary Fig. 6).

The number of total T cells (CD3+ cells) as well as the CD4+ and CD8+T cells in iLN were analyzed by flow cytometry. No differences between OA and control mice were detected at 2 weeks post-surgery (Supplementary Fig. 7A, B, and C) or 8 weeks post-surgery (Supplementary Fig. 7D, E, and F). The gating strategy for the T cell populations is shown in Supplementary Fig. 8.

### Estrogen prevents cartilage degradation and decreases synovial hyperplasia in mice with OA

To determine the influence of physiological estrogen levels on OA disease progression, mice were subjected to DMM surgery after OVX or sham operation and treated with vehicle (Veh) or E2 (0.15 µg) every 4 days for 8 weeks. The uterus is a highly estrogen-responsive tissue and measurement of the uterus weight is used to confirm successful OVX surgery and E2 replacement (Modder *et al.* 2004). As expected, the weight of the uterus drastically declined after OVX and increased after both 2 and 8 weeks of E2 treatment (Fig. 1C and D). Additionally, the serum levels of testosterone were decreased in OVX+Veh compared to Sham+Veh treated mice, measured 8 weeks after the beginning of the treatment (Fig. 1E).

The OARSI score revealed no difference in cartilage degradation in the OVX+Veh group compared to Sham+Veh, while E2 replacement partially prevented cartilage degeneration (Fig. 2A and C). The thickness of the synovial membrane did not differ between the OVX+Veh and Sham+Veh groups, but a significant decrease in synovial thickness was observed in OVX mice treated with E2 (Fig. 2B and D).

### Estrogen ameliorates locomotor activity and pain sensitivity 2 weeks after OA induction

The mice were subjected to motor tests 2 and 8 weeks after DMM surgery. At 2 weeks post-DMM surgery, OVX mice showed a tendency toward decreased movement compared to mice with intact ovaries (Fig. 3A, left). E2 treatment for 2 weeks increased both track length and velocity compared with OVX mice receiving Veh treatment. E2 treatment also enhanced the forced locomotor activity by increasing the time that the animals spent walking on the apparatus (Fig. 3B, left). At 8 weeks post-DMM surgery, neither OVX nor E2 treatment influenced the motor ability of the mice (Fig. 3A and B, right).

Mechanical allodynia was tested 2 weeks after DMM surgery. Alteration of the motor behavior in OVX+Veh mice at this time point was associated with decreased pain threshold (*i.e.* increased pain sensitivity) on the paw of the OA leg in the OVX+Veh group compared with Sham+Veh. Replacement with a physiological concentration of E2 increased the threshold for pain sensitivity in the paw of the OA leg (Fig. 3C, left). No differences between the groups were found for the paw on the contralateral leg (Fig. 3C, right).

### Estrogen partly changes the body composition of mice with OA

The body composition of mice with OA was analyzed by DXA. The body weight did not differ between vehicle-treated sham and OVX groups (Fig. 4A) despite increased fat percentage in OVX mice compared to sham (Fig. 4B). E2 treatment for 8 weeks slightly increased the body weight compared with OVX+Veh (Fig. 4A) but did not prevent the accumulation of fat tissue (Fig. 4B). Instead E2-treated mice displayed a slight increase in lean mass (Fig. 4C) compared to OVX+Veh.

A reduction of the BMC was shown in OA mice after OVX compared to mice with intact ovaries, while this effect was reversed by E2 treatment (Fig. 4D).

Scanning of the lumbar vertebrae (L3 and L4) showed a reduction of the aBMD in OVX+Veh compared to the Sham+Veh group (Fig. 4E). Treatment with E2 prevented the loss of aBMD from the lumbar spine (Fig. 4E).

### Estrogen inhibits OVX-induced trabecular but not cortical bone loss in mice with OA

In Sham+Veh and OVX+Veh mice, the articular surface appeared irregular and treatment with E2 improved the appearance of the articular surface (Fig. 5A). However, neither OVX nor E2 treatment affected the cortical bone volume value in OA mice at 8 weeks post-DMM surgery (Fig. 5B). Cortical BMD decreased significantly after OVX compared with sham, but treatment with E2 did not result in a significant increase of the BMD (Fig. 5C).

The trabecular bone volume and BMD in the subchondral area were significantly decreased in OVX+Veh compared to the Sham+Veh group, while E2 treatment did not affect BV/TV or BMD (Fig. 6A and B). OVX resulted in decreased trabecular thickness (Fig. 6C) and increased bone erosion (bone surface/bone volume, Fig. 6D) compared with sham, and E2 replacement prevented the decrease of trabecular thickness and diminished bone erosion in the subchondral area.

### Estrogen influences the number of T cells in the iLN of mice with OA

Cells from iLN were isolated at 2 and 8 weeks after DMM surgery and analyzed by flow cytometry for T cell populations in sham and OVX mice treated with Veh or E2. OVX resulted in an increase of total as well as CD4+ and CD8+ T cells in iLN, while E2 treatment for 2 weeks had no effect on the number of T cells (Fig. 7A, B and C). In contrast, at 8 weeks post-DMM surgery, the number of T cells in iLN did not differ between Sham+Veh and OVX+Veh, while 8 weeks treatment of OVX mice with E2 significantly decreased the number of total as well as CD4+ and CD8+ T cells in iLN (Fig. 7D and E).

## Discussion

The purpose of this study was to evaluate the role of female sex steroid deficiency and E2 replacement to OVX mice on OA progression. OVX mice subjected to an experimental model of OA were treated with a physiological dose of E2 in an early phase of the OA disease progression (Byers *et al.* 2012). E2 was administered in a pulsed fashion to resemble normal hormone fluctuations during the murine estrous cycle (Corciulo *et al.* 2021).

In this study, OVX of mice subjected to OA only resulted in a nonsignificant tendency towards increased cartilage degradation and synovial thickness compared to OA mice with intact ovaries. However, E2 replacement prevented proteoglycan loss and fibrillation of the articular surface as well as the thickening of the synovium, suggesting an important protective role for this hormone in joint tissues. Similarly, Moritake and coworkers described osteopenia of the subchondral bone after OVX in mice subjected to OA, and no differences in the cartilage appearance and OARSI score between sham-operated and OVX mice (Moritake *et al.* 2017). Testosterone has been negatively linked to OA progression (Ma *et al.* 2007). In this study, the levels of testosterone were significantly decreased in serum from OVX+Veh mice compared to Sham+Veh. It is possible that the decline in testosterone compensates for the loss of estradiol after OVX in mice subjected to OA, resulting in the lack of difference in OARSI score and synovial thickness between sham and OVX mice with OA.

In contrast to our results, previous studies have demonstrated worse OA outcomes in OVX animals compared to sham-operated controls (Ge *et al.* 2019). In a model of postmenopausal OA in rats, OVX surgery accelerated cartilage and bone turnover, an effect that could be inhibited by the administration of estrogen (Xu *et al.* 2019) and ER modulators (Hoegh-Andersen *et al.* 2004). Additionally, OVX C3H/HeJ mice displayed an augmented OA phenotype compared to mice with intact ovaries (Sniekers *et al.* 2010). This difference could be due to the animal model used (iodoacetate-induced OA), i.e. estrogen deprivation could be more detrimental in an OA model where a stronger pro-inflammatory component is used. Residues of phytoestrogens in the chow could also influence the results in OVX mice. Moreover, we cannot exclude the possibility that differences between sham and OVX mice with OA could be increased at a later time point of the OA disease progression.

In this study, OA mice showed increased lumbar spine aBMD compared to controls. A similar effect has been found in human subjects with knee OA. In these patients, knee OA with a low radiographic score was associated with increased BMD in the vertebrae. On the contrary, in OA patients with a high radiographic score, signs of osteoporosis were found in the vertebrae (Kim *et al.* 2018). In mice, the DMM, and consequently the destabilization of the whole joint, leads to alteration of the gait (Alves *et al.* 2020). This could result in stress on the lumbar spine with an increased BMD as a consequence in this early phase of the disease progression.

Like in human OA, experimental models of OA also result in alterations of the subchondral bone. The subchondral bone has the important function to absorb mechanical shock, dynamically adjusting the orientation of the trabeculae, and to provide nutrients for the adjacent articular cartilage (Li *et al.* 2013). Subchondral sclerosis and the presence of osteophytes are hallmarks for OA and result from a process of endochondral ossification. As a consequence of this fast turnover, the bone increases its volume without appropriate mineralization resulting in an increased bone volume and an osteoporotic BMD (Pauly *et al.* 2015, Goldring & Goldring 2016). Estrogens have been shown to regulate bone turnover directly by binding to ERs on osteoblasts and osteoclasts, and indirectly by regulating T cells in an inflammatory setting (Cenci *et al.* 2000, Roggia *et al.* 2001, Khosla *et al.* 2012, Vanderschueren *et al.* 2014). Removal of the ovaries and the resulting reduction of estrogen levels lead to osteoporosis of the cortical and trabecular subchondral bone. In OA, this worsens the condition of the articular cartilage. In our experimental model, estrogen replacement prevented OVX-induced loss of trabecular bone but did not affect the cortical bone. These results differ from the study by Sniekers and coworkers where an effect of estradiol was also shown on the subchondral cortical bone in mice with OA induced by iodoacetate (Sniekers *et al.* 2008). The discordant results could be due to differences in the mouse strain, the OA model, or the higher E2 dose used by Sniekers and coworkers (12 µg/day by subcutaneously implanted pellets) (Sniekers *et al.* 2008).

In the last decades, new knowledge on OA shifted the paradigm of OA as a ‘wear and tear’ disease towards the recognition of an important inflammatory component that, although mild and localized around the damaged area, characterizes and drives the progression of the disease (Scanzello 2017, van den Bosch 2019). No signs of inflammation were detected in the peripheral blood of OA patients. Instead, infiltrated CD4+ T cells were found in the synovial membrane, at frequencies that increased with the severity of the disease (Moradi *et al.* 2014). CD8+ T cells were also increased in OA patients with higher radiographic grading (Apinun *et al.* 2016). In our experiment terminated after 2 weeks, OVX resulted in increased numbers of both CD4+ and CD8+ T cells in iLN, the lymph node draining the hind leg subjected to OA. Additionally, treatment of OVX mice with E2 for 8 weeks significantly decreased T cells in iLN, indicating a local anti-inflammatory effect induced by the long E2 treatment.

Pain in OA is caused by an inflammatory and a neurogenic component. In this study, the motor activity of OA mice was slightly decreased in the group subjected to OVX, an effect that was associated with increased pain sensitivity. The motor activity improved by E2 replacement for 2 weeks, which was also associated with lower pain sensitivity of the OA paw. OVX has previously been associated with inactivity in mice and treatment with E2 stimulated movement and reduced hyperalgesia (Gorzek *et al.* 2007, Sanoja & Cervero 2008, Cabelka *et al.* 2019, Chen *et al.* 2021). Interestingly, previous work shows that pain in mice subjected to the DMM model starts at 4 weeks after the surgery and stays stable until the last time point analyzed at 16 weeks. However, in that study, neither female mice nor earlier time points were analyzed (Miller *et al.* 2012). A recent study by Hwang and colleagues shows that pain is detected by using the von Frey test starting 2 weeks after DMM surgery (Hwang *et al.* 2021). The reduction of pain after E2 treatment for 2 weeks in this study could be a consequence of a faster resolution of a DMM surgery-related inflammatory component in the presence of E2 or could be due to the stimulation of ERs in the dorsal root ganglion, which is responsible for mediating the transmission of pain information to the brain (Mowa *et al.* 2003). Future experiments with a longer duration are warranted to evaluate differences in motor ability and pain behavior between the groups.

## Conclusion

Our study demonstrates that the removal of ovarian hormones is not a trigger of the disease but instead a facilitator of the OA progression since both bone parameters and T cell numbers are altered by OVX. Thus, in OVX mice, the diminished subchondral bone mineralization and mild immune activation limit the healing processes necessary for spontaneous cartilage regeneration and lead to worsening of the disease progression. In this study, we also clarified that a physiological dose of E2 administrated in a pulsed fashion improves OA symptoms.

## Supplementary Material

Supplementary figure 1 - Uterus weight and testosterone measurement. Uterus weight was recorded in mice subjected to DMM (OA group) or control surgery (Control group), sacrificed after two weeks (A) and eight weeks (B). The amount of testosterone was measured in serum after eight weeks (C). Data are expressed as mean±SD and analyzed by t-test. *p<0.05, ns = not statistically significant.

Supplementary figure 2 - Cartilage degradation and fibrillation, but not synovial hyperplasia, are visible eight weeks after DMM surgery. Mice subjected to surgery for destabilization of the medial meniscus (OA group) or control surgery (Control group) were sacrificed after eight weeks. The knees were collected for histological assessment. Representative images of the knee articular surfaces stained with the safranin-O/fast green staining (A, scale bar = 500 µm) and plotted OARSI score quantification (B). Representative figures of H&E staining of the knee joint (C, 10x magnification on the top section, 20x magnification on the bottom section, scale bar = 500 µm) and quantification of the synovial thickness (synovial area/perimeter; D). Data are expressed as mean±SD and analyzed by t-test. **p<0.01, ns = not statistically significant.

Supplementary figure 3 – OA mice do not show a deterioration of motor ability and pain sensitivity at an early stage of the disease progression. Mice were subjected to surgery for destabilization of the medial meniscus (OA group) or control surgery (Control group) and were engaged in motor tests (two and eight weeks after surgery) and pain tests (two weeks after surgery). The graphs show the track length and the velocity of the experimental mice in the arena of the open field test (A) and the latency to fall from the rotarod apparatus at the two time points (B). Data from the Von Frey test show the paw withdrawal threshold for the OA and the contralateral leg of the experimental animals (C). Data are expressed as mean±SD and analyzed by t-test. ns = not statistically significant.

Supplementary figure 4 – OA mice do not differ in body composition at an early stage of the disease compared to control mice. Mice subjected to surgery for destabilization of the medial meniscus (OA group) or control surgery (Control group) were sacrificed after eight weeks. DXA scan was performed before sacrifice and lumbar vertebrae were collected for µCT analysis. The graphs describe the total body weight (A), DXA measurements of the body fat percentage (B), lean weight (C), total body bone mineral content (BMC) (D), lumbar spine (LS) areal bone mineral density (aBMD) measured by DXA (E). Data are expressed as mean±SD and analyzed by using t-test. *p<0.05, ns = not statistically significant.

Supplementary figure 5 – OA mice show increased bone volume of the cortical subchondral area. Mice subjected to surgery for destabilization of the medial meniscus (OA group) or control surgery (Control group) were sacrificed after eight weeks. Knees were collected for µCT analysis. Representative pictures of 3D knee joints reconstruction from µCT data (A). The yellow rectangles highlight the areas of bone loss and pittings of the articular surface. The red circles delimit the area where the irregular articular bone surface is visible. The graphs contain the plotted data from the µCT analysis of the cortical bone volume/tissue volume (BV/TV; B) and the cortical bone mineral density (BMD; C). Data are expressed as mean±SD and analyzed by t-test. ****, p<0.0001, ns = not statistically significant.

Supplementary figure 6 – No alterations of the subchondral trabecular bone are detected in OA mice at an early stage of the disease. Mice subjected to surgery for destabilization of the medial meniscus (OA group) or control surgery (Control group) were sacrificed after eight weeks. Knees were collected for µCT analysis. The graphs display the plotted data from the µCT analysis of the trabecular subchondral bone volume/tissue volume (BV/TV; A), trabecular bone mineral density (BMD; B), trabecular (Trab.) thickness (C) and bone surface/bone volume (bone erosion; D). Data are expressed as mean±SD and analyzed by t-test. ns = not statistically significant.

Supplementary figure 7 – The number of CD4+ T cells and CD8+ T cells in inguinal lymph nodes are not affected in OA mice at an early stage of the disease. Mice subjected to surgery for destabilization of the medial meniscus (OA group) or control surgery (Control group) were sacrificed after two and eight weeks, and the inguinal lymph nodes were collected for FACS analysis. The graph shows the plotted data from the flow-cytometry analysis of total T cells, CD4+ T cells and CD8+ T cells at two weeks (A-C) and eight weeks after surgery (D-F). Data are expressed as mean±SD and analyzed by t-test; ns = not statistically significant.

Supplementary figure 8 – Gating strategy for the FACS analysis.

## Declaration of interest

The authors declare that there is no conflict of interest that could be perceived as prejudicing the impartiality of the research reported.

## Funding

This work was supported by grants from the Swedish Research Council (2016-01192, 2020-01885, 2020-02527), the Novo Nordisk Foundation (19928), the Swedish state under the agreement between the Swedish government and the county councils, the ALF-agreement (ALFGBG-716421, ALFGBG-965238), the Marie Sklodowska Curie Action (893560), the Association against Rheumatism (R-929920, R-940520, R-940384), King Gustav V’s 80 years’ foundation (FAI-2019-0573), the Nanna Svartz foundation (2020-00359), the Emil and Wera Cornells foundation, the Åke Wiberg Foundation (M20-0155), the Tore Nilsson Foundation, and the IngaBritt and Arne Lundberg Foundation (LU2018-0008, LU2020-0010).
