# Supplementary material for: Physiological levels of estradiol limit murine osteoarthritis progression
Source: J Endocrinol. 2022 Aug 16;255(2):39–51. doi: 10.1530/JOE-22-0032 (PMC9513658; doi:10.1530/JOE-22-0032)
Supplement: Supplementary figure 1 - Uterus weight and testosterone measurement. Uterus weight was recorded in mice subjected to DMM (OA group) or control surgery (Control group), sacrificed after two weeks (A) and eight weeks (B). The amount of testosterone was measured in serum after eight weeks (C). Data are [file supplementary_figure_1.pdf]

**Supplementary figure 1**

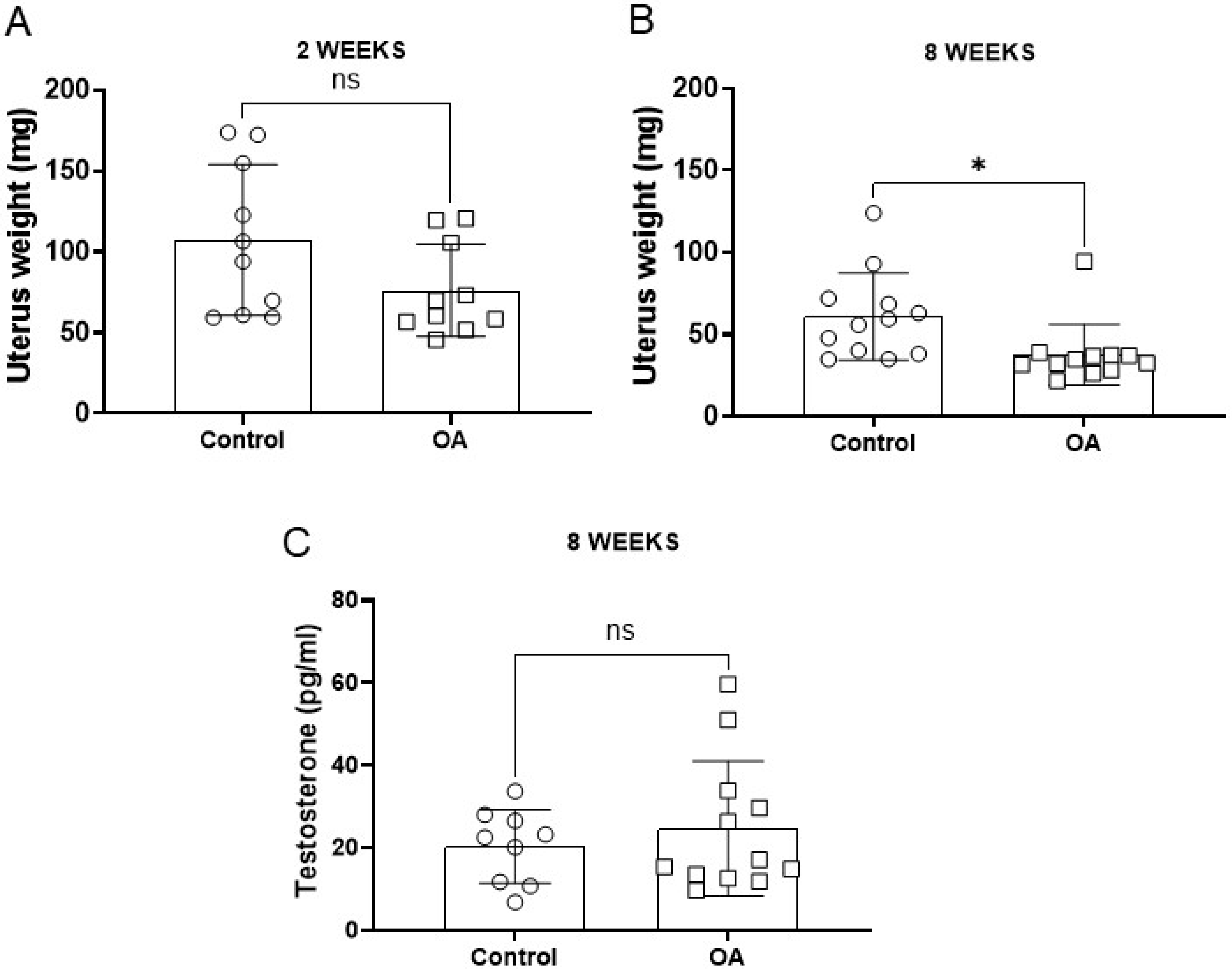


Supplementary figure 1 - **Uterus weight and testosterone measurement**. Uterus weight was recorded in mice subjected to DMM (OA group) or control surgery (Control group), sacrificed after two weeks (A) and eight weeks (B). The amount of testosterone was measured in serum after eight weeks (C). Data are expressed as mean±SD and analyzed by t-test. *$p<0.05$, ns = not statistically significant.
