# Supplementary material for: Physiological levels of estradiol limit murine osteoarthritis progression
Source: J Endocrinol. 2022 Aug 16;255(2):39–51. doi: 10.1530/JOE-22-0032 (PMC9513658; doi:10.1530/JOE-22-0032)
Supplement: Supplementary figure 2 - Cartilage degradation and fibrillation, but not synovial hyperplasia, are visible eight weeks after DMM surgery. Mice subjected to surgery for destabilization of the medial meniscus (OA group) or control surgery (Control group) were sacrificed after eight weeks. The knees we [file supplementary_figure_2.pdf]

Supplementary figure 2

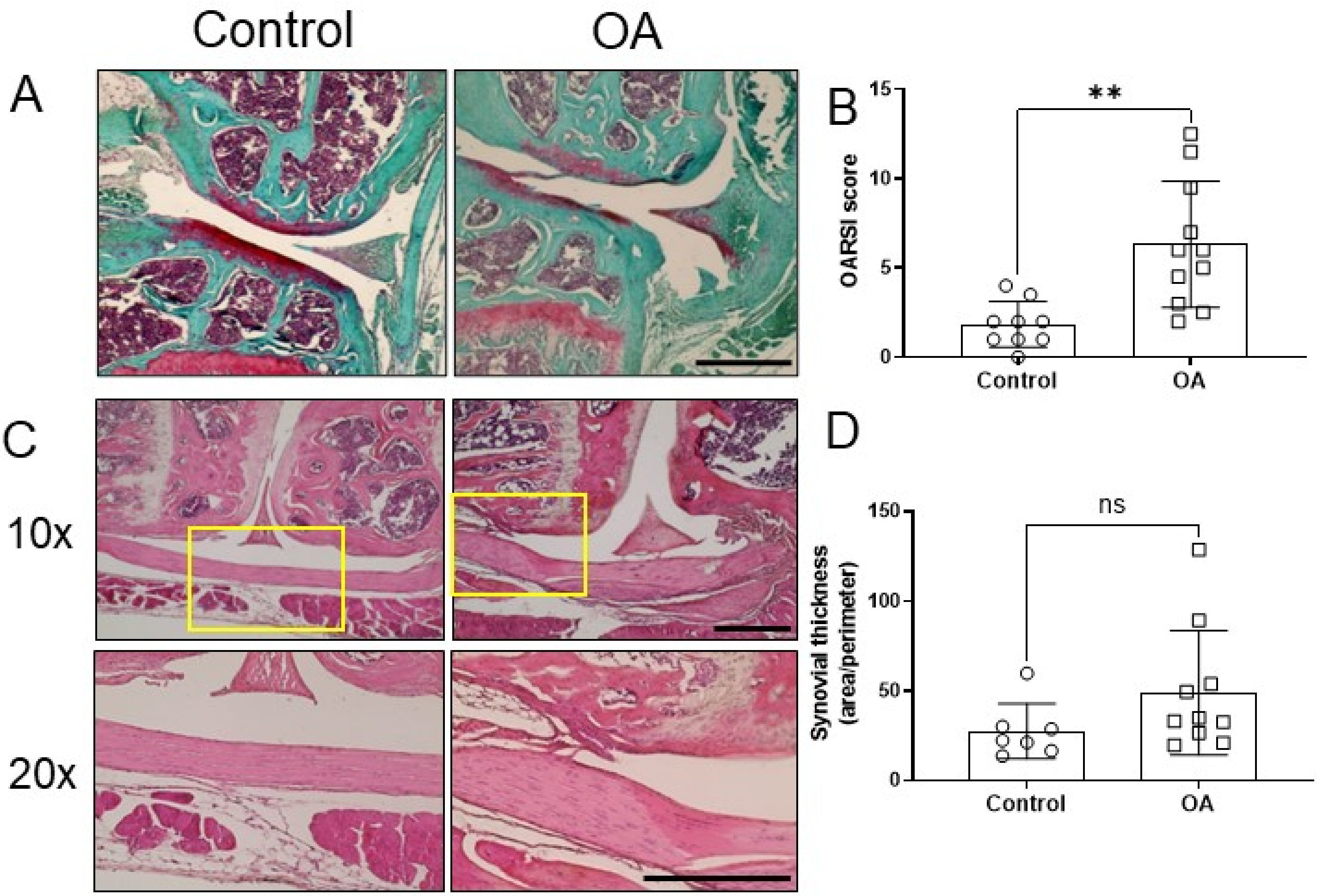

Supplementary figure 2 - **Cartilage degradation and fibrillation, but not synovial hyperplasia, are visible 8 weeks after DMM surgery.** Mice subjected to DMM (OA group) or control surgery (Control group) were sacrificed after 8 weeks. The knees were collected for histological assessment. Representative images of the knee articular surfaces stained with the safranin-O/fast green staining (A, scale bar = 500 µm) and plotted OARSI score quantification (B). Representative figures of H&E staining of the knee joint (C, 10x magnification on the top section, 20x magnification on the bottom section, scale bar = 500 µm) and quantification of the synovial thickness (synovial area/perimeter; D). Data are expressed as mean±SD and analyzed by t-test. **$p<0.01$, ns = not statistically significant.
