# Supplementary material for: Physiological levels of estradiol limit murine osteoarthritis progression
Source: J Endocrinol. 2022 Aug 16;255(2):39–51. doi: 10.1530/JOE-22-0032 (PMC9513658; doi:10.1530/JOE-22-0032)
Supplement: Supplementary figure 3 – OA mice do not show a deterioration of motor ability and pain sensitivity at an early stage of the disease progression. Mice were subjected to surgery for destabilization of the medial meniscus (OA group) or control surgery (Control group) and were engaged in motor tests (tw [file supplementary_figure_3.pdf]

Supplementary figure 3

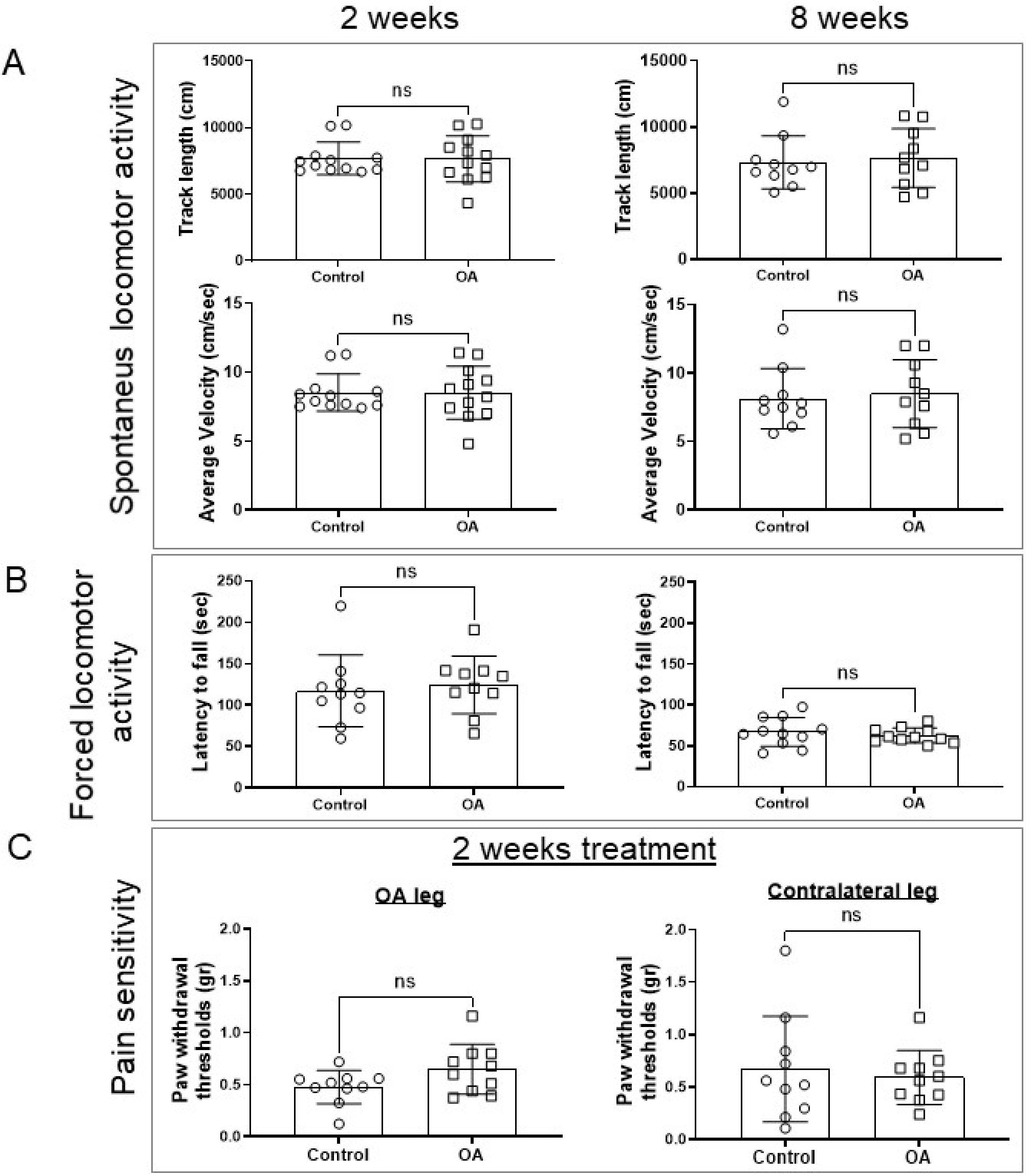

Supplementary figure 3 – **OA mice do not show a deterioration of motor ability and pain sensitivity at an early stage of the disease progression.** Mice were subjected to DMM (OA group) or control surgery (Control group) and were engaged in motor tests (2 and 8 weeks after surgery) and pain tests (2 weeks after surgery). The graphs show the track length and the velocity of the experimental mice in the arena of the open field test (A) and the latency to fall from the rotarod apparatus at the two time points (B). Data from the Von Frey test show the paw withdrawal threshold for the OA and the contralateral leg of the experimental animals (C). Data are expressed as mean±SD and analyzed by t-test. ns = not statistically significant.
