# Supplementary material for: Physiological levels of estradiol limit murine osteoarthritis progression
Source: J Endocrinol. 2022 Aug 16;255(2):39–51. doi: 10.1530/JOE-22-0032 (PMC9513658; doi:10.1530/JOE-22-0032)
Supplement: Supplementary figure 4 – OA mice do not differ in body composition at an early stage of the disease compared to control mice. Mice subjected to surgery for destabilization of the medial meniscus (OA group) or control surgery (Control group) were sacrificed after eight weeks. DXA scan was performed b [file supplementary_figure_4.pdf]

Supplementary figure 4

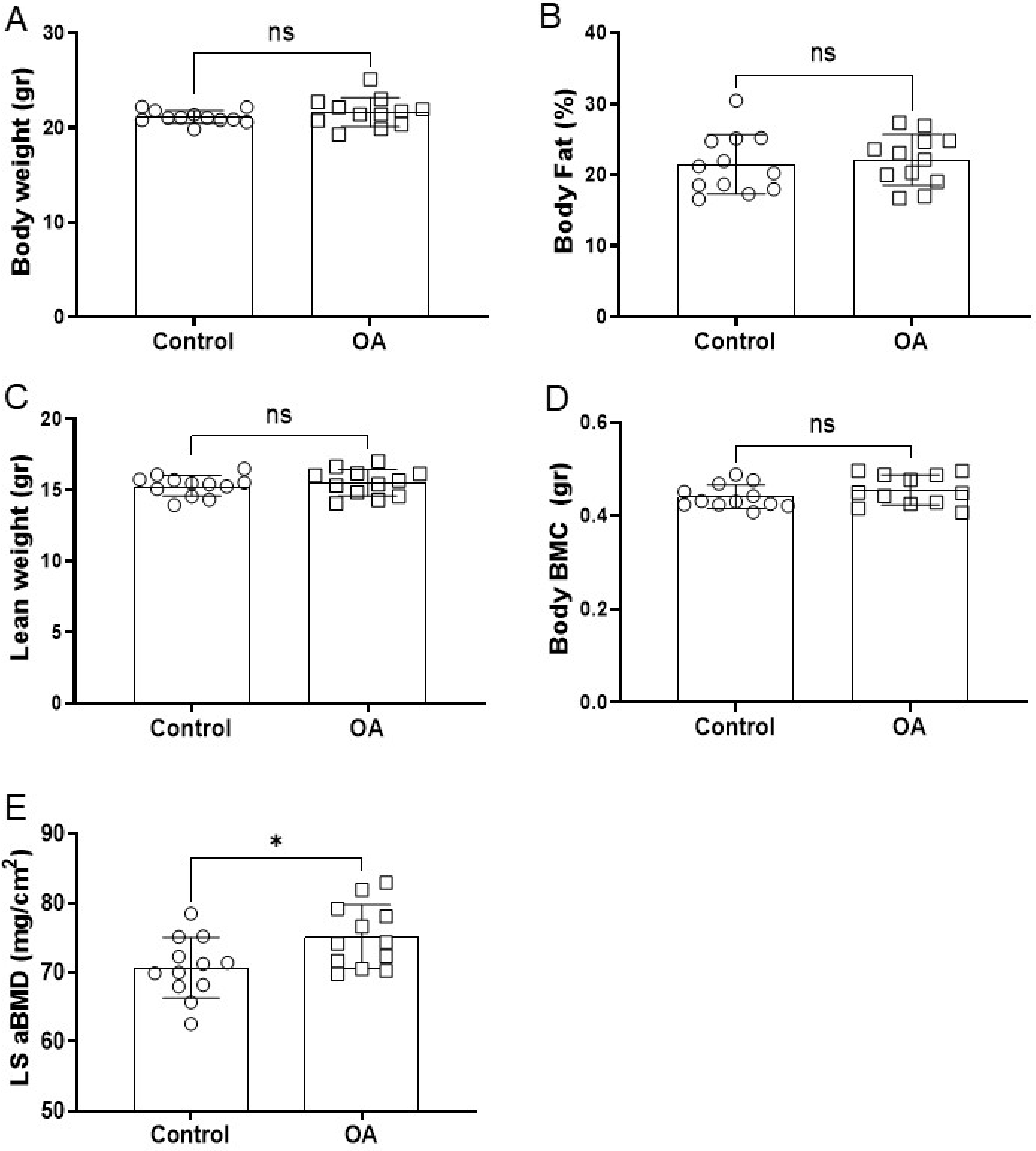

Supplementary figure 4 – **OA mice do not differ in body composition at an early stage of the disease compared to control mice.** Mice subjected to DMM (OA group) or control surgery (Control group) were sacrificed after 8 weeks. DXA scan was performed before sacrifice and lumbar vertebrae were collected for μCT analysis. The graphs describe the total body weight (A), DXA measurements of the body fat percentage (B), lean weight (C), total body bone mineral content (BMC) (D), lumbar spine (LS) areal bone mineral density (aBMD) measured by DXA (E). Data are expressed as mean±SD and analyzed by using t-test. *$p<0.05$, ns = not statistically significant.
