# Supplementary material for: Physiological levels of estradiol limit murine osteoarthritis progression
Source: J Endocrinol. 2022 Aug 16;255(2):39–51. doi: 10.1530/JOE-22-0032 (PMC9513658; doi:10.1530/JOE-22-0032)
Supplement: Supplementary figure 5 – OA mice show increased bone volume of the cortical subchondral area. Mice subjected to surgery for destabilization of the medial meniscus (OA group) or control surgery (Control group) were sacrificed after eight weeks. Knees were collected for µCT analysis. Representative pi [file supplementary_figure_5.pdf]

Supplementary figure 5

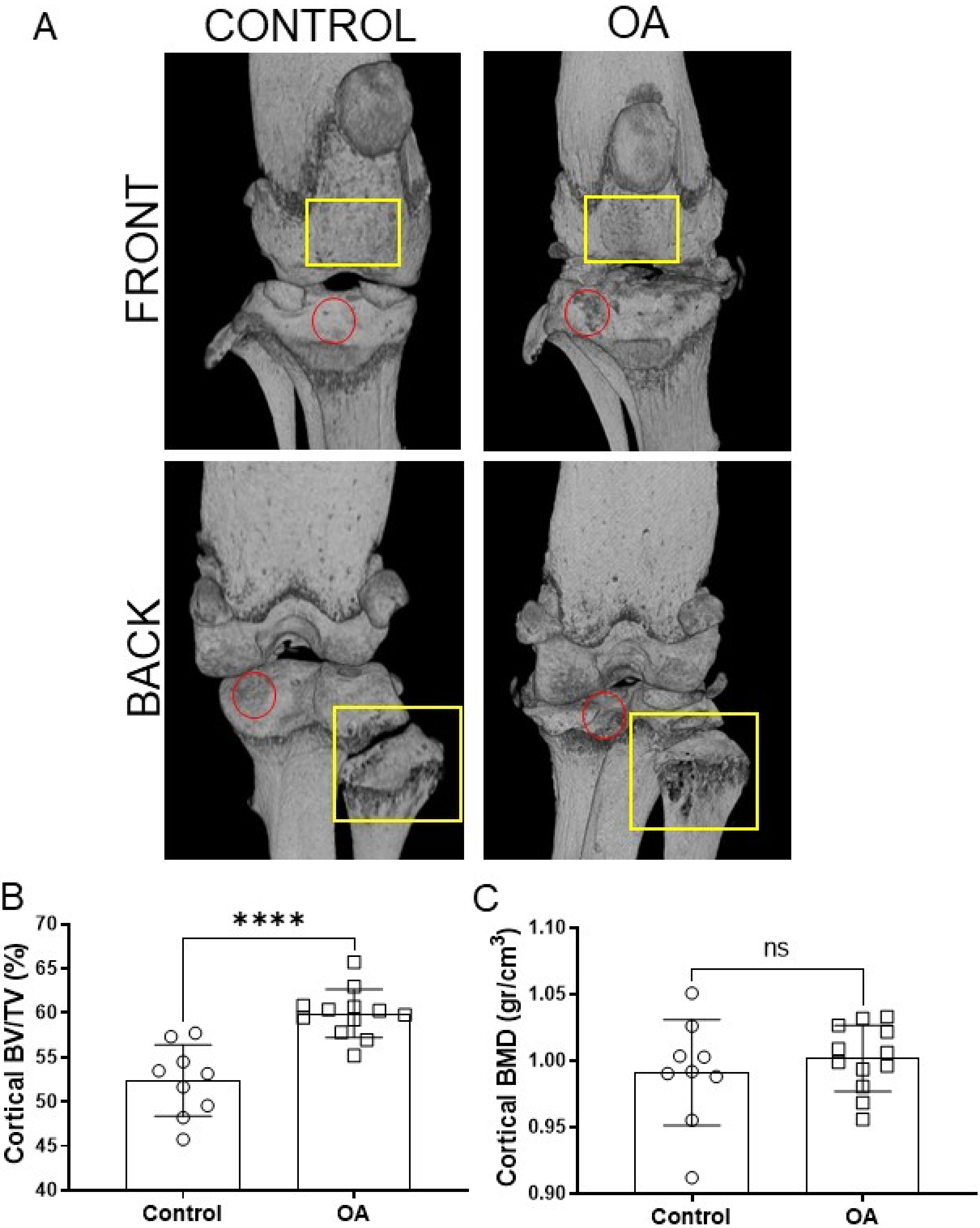

Supplementary figure 5 – **OA mice show increased bone volume of the cortical subchondral area.** Mice subjected to DMM (OA group) or control surgery (Control group) were sacrificed after 8 weeks. Knees were collected for µCT analysis. Representative pictures of 3D knee joints reconstruction from µCT data (A). The yellow rectangles highlight the areas of bone loss and pittings of the articular surface. The red circles delimits the area where irregular articular bone surface is visible. The graphs contains the plotted data from the µCT analysis of the cortical bone volume/tissue volume (BV/TV; B) and the cortical bone mineral density (BMD; C). Data are expressed as mean±SD and analyzed by t-test. ****, $p<0.0001$, ns = not statistically significant.
