# Supplementary material for: Physiological levels of estradiol limit murine osteoarthritis progression
Source: J Endocrinol. 2022 Aug 16;255(2):39–51. doi: 10.1530/JOE-22-0032 (PMC9513658; doi:10.1530/JOE-22-0032)
Supplement: Supplementary figure 6 – No alterations of the subchondral trabecular bone are detected in OA mice at an early stage of the disease. Mice subjected to surgery for destabilization of the medial meniscus (OA group) or control surgery (Control group) were sacrificed after eight weeks. Knees were collec [file supplementary_figure_6.pdf]

Supplementary figure 6

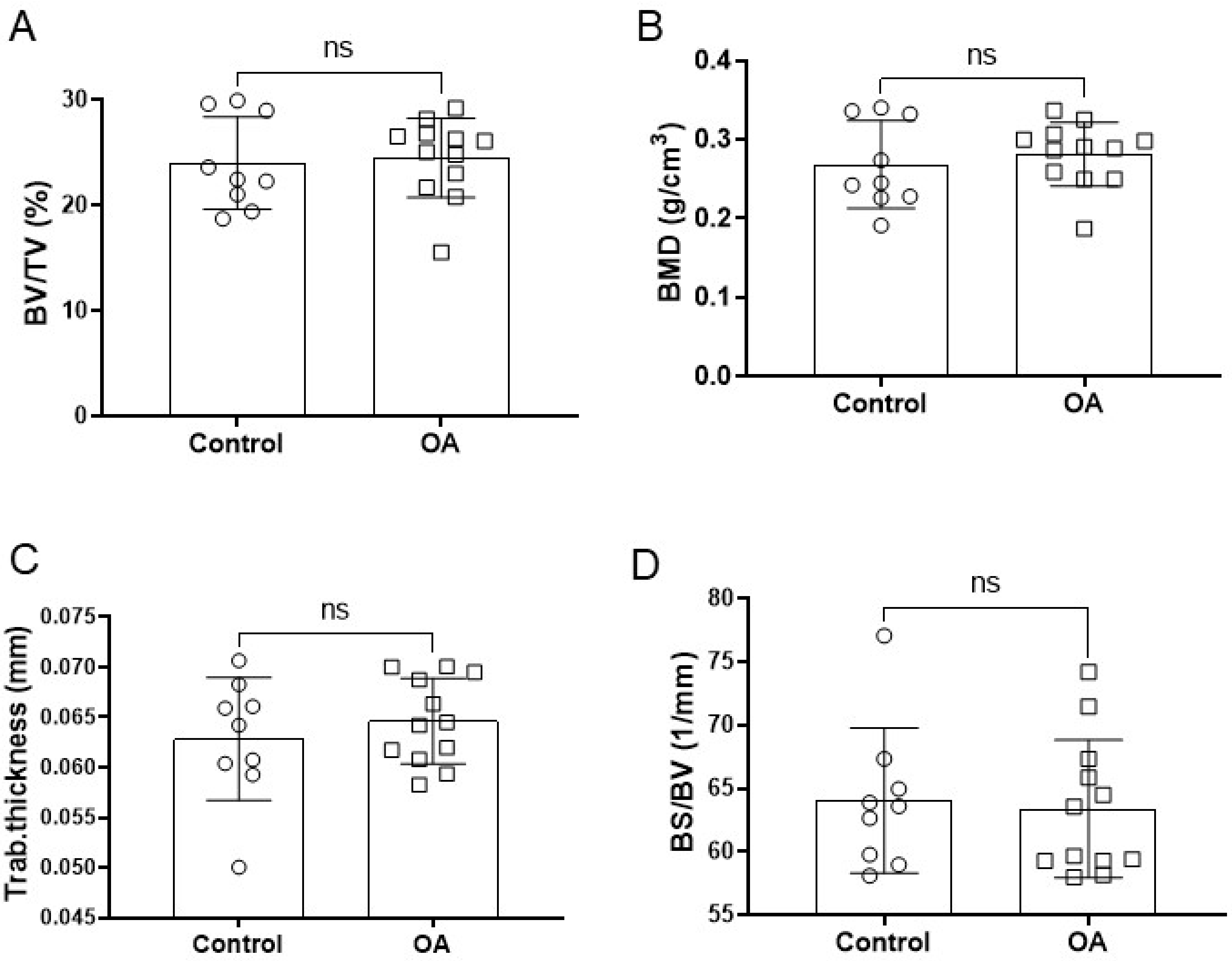

Supplementary figure 6 – **No alterations of the subchondral trabecular bone are detected in OA mice at an early stage of the disease.** Mice subjected to DMM (OA group) or control surgery (Control group) were sacrificed after 8 weeks. Knees were collected for µCT analysis. The graphs display the plotted data from the µCT analysis of the trabecular subchondral bone volume/tissue volume (BV/TV; A), trabecular bone mineral density (BMD; B), trabecular (Trab.) thickness (C) and bone surface/bone volume (bone erosion; D). Data are expressed as mean±SD and analyzed by t-test. ns = not statistically significant.
