# Supplementary material for: Physiological levels of estradiol limit murine osteoarthritis progression
Source: J Endocrinol. 2022 Aug 16;255(2):39–51. doi: 10.1530/JOE-22-0032 (PMC9513658; doi:10.1530/JOE-22-0032)
Supplement: Supplementary figure 7 – The number of CD4+ T cells and CD8+ T cells in inguinal lymph nodes are not affected in OA mice at an early stage of the disease. Mice subjected to surgery for destabilization of the medial meniscus (OA group) or control surgery (Control group) were sacrificed after two and  [file supplementary_figure_7.pdf]

Supplementary figure 7

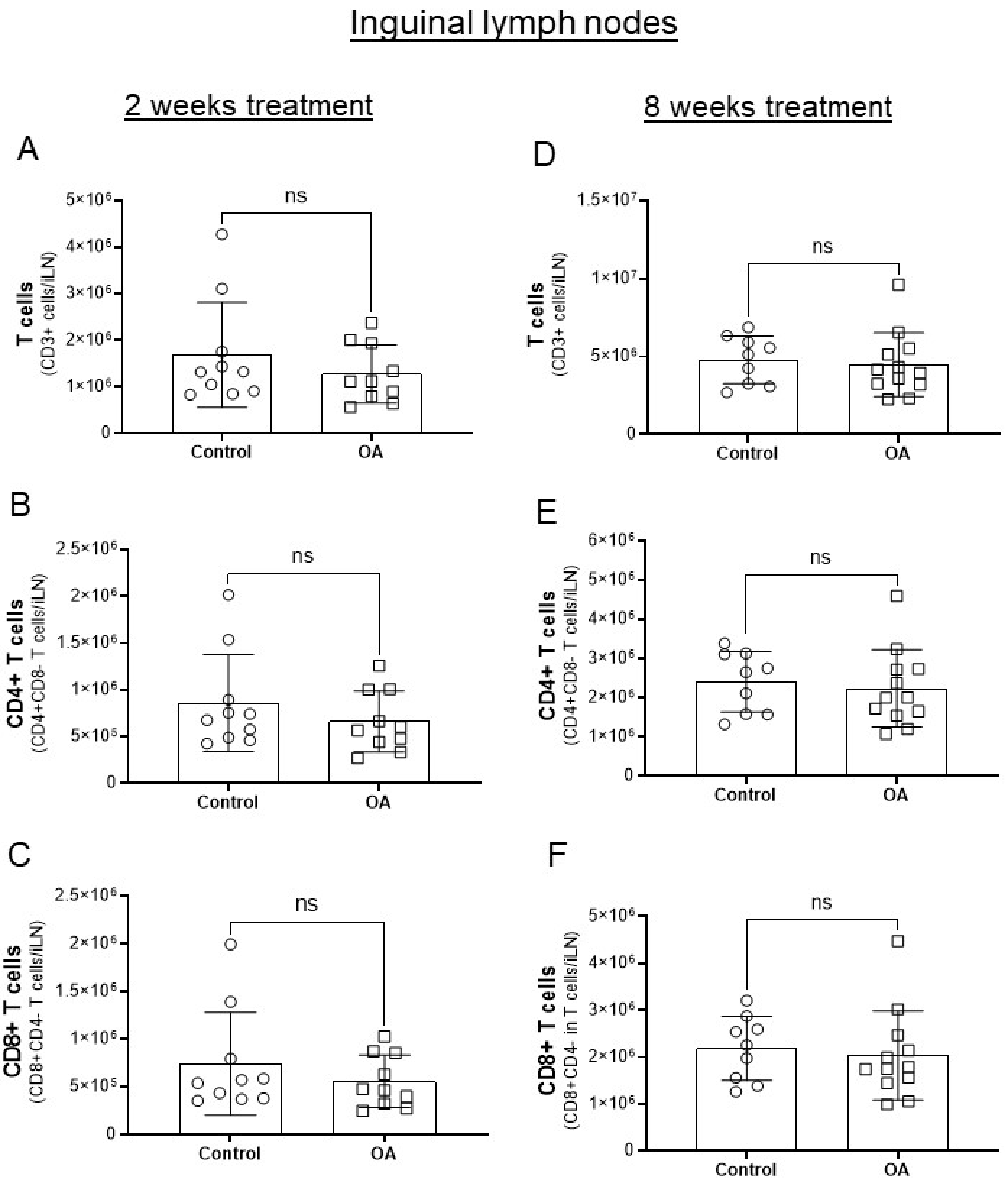

Supplementary figure 7 – **The numbers of CD4+ T cells and CD8+ T cells in inguinal lymph nodes are not affected in OA mice at an early stage of the disease.** Mice subjected to DMM (OA group) or control surgery (Control group) were sacrificed after 2 and 8 weeks, and the inguinal lymph nodes were collected for FACS analysis. The graphs show the plotted data from the FACS analysis of total T cells, CD4+ T cells and CD8+ T cells at 2 weeks (A-C) and 8 weeks after surgery (D-F). Data are expressed as mean±SD and analyzed by t-test; ns = not statistically significant.
