# Supplementary material for: Physiological levels of estradiol limit murine osteoarthritis progression
Source: J Endocrinol. 2022 Aug 16;255(2):39–51. doi: 10.1530/JOE-22-0032 (PMC9513658; doi:10.1530/JOE-22-0032)
Supplement: Supplementary figure 8 – Gating strategy for the FACS analysis. [file supplementary_figure_8.pdf]

Supplementary figure 8

Gating strategy

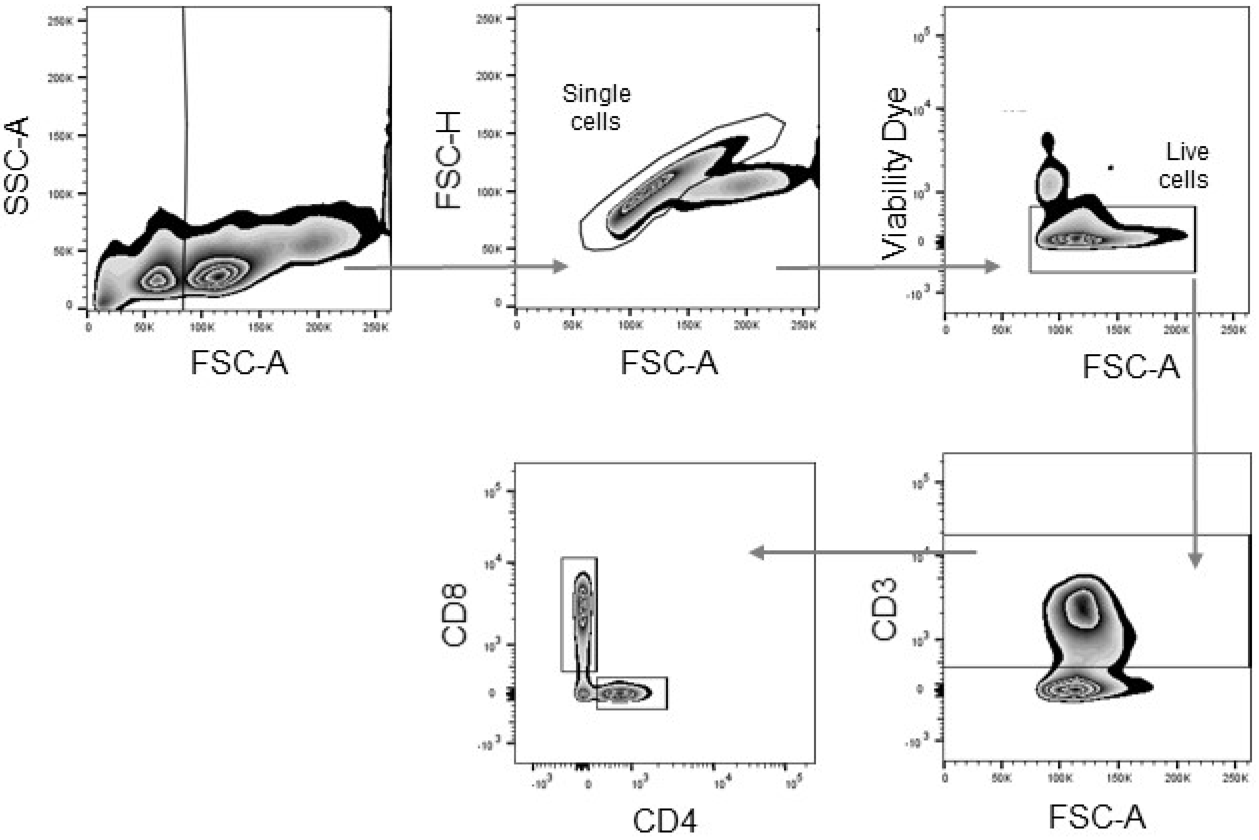

Supplementary figure 8 – **Gating strategy for the FACS analysis.**
